# Percutaneous restoration of bone continuity with screws and PMMA cement in an extensive destruction of the pelvis

**DOI:** 10.1051/sicotj/2019011

**Published:** 2019-05-22

**Authors:** Anne-Laure Hermann, Charles Pioger, Claudia Rizzo, Guillaume Odri, Jean-Denis Laredo

**Affiliations:** 1 Department of Radiology, Hôpital Lariboisière, Assistance Publique des Hôpitaux de Paris (APHP) 75010 Paris France; 2 Department of Orthopedic Surgery, Hôpital Lariboisière, Assistance Publique des Hôpitaux de Paris (APHP) 75010 Paris France; 3 Department of Oncological, Centre Hospitalier René Dubos 95300 Pontoise France; 4 Université Paris Diderot, Sorbonne Paris Cité, Faculté de Médecine 75013 Paris France

**Keywords:** Combined fixation, Percutaneous stabilization, Cementoplasty, Screwing

## Abstract

We report a case of combined percutaneous screw placement and cementoplasty guided by CT and fluoroscopy in a 66-year-old man with extensive osteolytic destruction of the right iliac bone and sacral wing due to metastasic infiltrative vesical carcinoma. The medical condition was responsible for very limited and painful walking. Two perpendicular screws were inserted into the iliac bone and sacroiliac joint, and bone cement injection was used to anchor the screws and restore the mechanical continuity of the pelvis ring. This minimally invasive procedure allowed for significant and rapid resumption of painless walking.

## Introduction

Bone metastasis develops in approximately 80% of patients with advanced cancer [1]. In addition to systemic treatments, local treatments such as radiation therapy, cementoplasty (percutaneous intraosseous injection of polymethylmetacrylate [PMMA] cement) or surgery may be needed to control pain and restore function. Cementoplasty is efficient in decreasing pain and restoring bone strength for bones submitted to compressive forces such as the vertebral body or acetabulum [2,3]. However, in skeletal areas submitted to shearing forces such as the pelvis, metal fixation is required to restore a mechanically competent bone. Surgical fixation is usually efficient to restore bone mechanical competence in long bone metastases. However, with extensive destruction of the pelvic bones, such as with metastasis, surgical reconstruction implies increased risk of failure, complications, and delay of systemic chemotherapy or radiotherapy [4]. In addition, many patients with advanced metastatic disease are usually poor surgical candidates owing to their general condition and short life expectancy.

Percutaneous interventions combining nailing or screw placement with cementoplasty are well-established methods for treating bone metastases with fractures or at high risk of pathological fracture, especially in older patients with comorbidities. Such interventions have been used to stabilize bone metastases of the femoral neck [5,6], other long bones of the lower limb [7], the spine [8], and the iliac bone [9]. A recent retrospective study reported promising results with this method for 34 metastatic bone lesions located in the spine, femur, and iliac bone, with rapid pain relief and good recovery of walking ability [10]. However, few authors have reported the use of this technique for extensive osteolysis of the pelvis involving both the iliac bone and sacrum [11].

We report a case of massive unilateral destruction of the pelvic bones due to metastasis that was treated percutaneously by placement of two screws, in the iliac bone and through the sacroiliac joint, combined with cementoplasty to create a bridge of PMMA cement between the two screws and reinforce osteosynthesis.

## Case report

**Figure 1:**
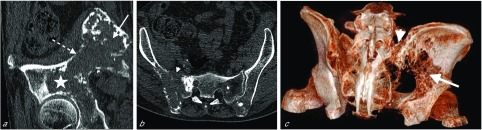
(a) Oblique sagittal reformatted view of pre-treatment CT (bone window, slice thickness 1 mm) of the iliac bone shows the extensive lesion involving the iliac wing (arrow), arcuate line (dotted arrow) and acetabulum (star). (b) Oblique axial reformatted view of pre-treatment CT (bone window, slice thickness 1 mm) of the pelvic bones shows the extensive lesion involving both the iliac bone and the sacral wing (arrowhead). (c) 3D oblique posterior reformatted view of the pelvic bones shows the extensive osteolytic lesion involving the iliac wing (arrow) and the sacral wing (arrowhead) greatly compromising pelvis stability.

A 66-year-old man with a history of infiltrative vesical carcinoma treated with intravesical immunotherapy 13 years previously was referred for recent-onset pain in the pelvic girdle. Pain was assessed by a visual analog scale (VAS) at 8/10 and was much increased with walking, which was limited to a short distance and required the use of a crutch. CT revealed extensive osteolytic metastasis involving almost all the right iliac wing and acetabulum together with the right sacral wing **(** Figure 1). Bone scintigraphy and ^18^F-FDG PET/CT revealed multiple other skeletal lesions. Biopsy of the pelvic bone revealed an epidermoid carcinoma. A vesical biopsy gave negative results, but because the prostate was clinically enlarged and the prostate-specific antigen level was increased (5 ng/mL), a prostate biopsy was performed and revealed epidermoid urothelial tumoral infiltration. The patient received a first course of systemic chemotherapy with carboplatine (AUC 5) and paclitaxel (80 mg/m^2^), a first infusion of bisphosphonates (4 mg acid zoledronic), and analgesics.

Given the pending risk of fracture and severe pain, the decision was taken in a multidisciplinary consultation to stabilize the pelvis before continuing systemic treatment and to attempt percutaneous osteosynthesis rather than open surgery because of the comorbidities and risk of complications with extensive surgery. Extension of the bone destruction to the sacral wing and anterior acetabulum precluded the use of the locked and perforated nail recently described to reinforce the arcuateiliac line [9]. Therefore, a combination of double screwing and cementoplasty was planned to restore weight-bearing force transmission from the spine to the hip.

**Figure 2:**
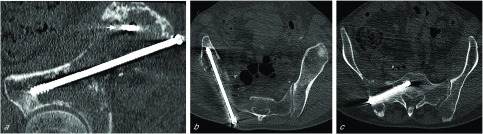
(a) Oblique sagittal reformatted view of the pelvis CT scan (bone window, slice thickness 1 mm) acquired after osteosynthesis (first step of the procedure). The first screw was placed into the iliac bone from the posterior iliac crest to the anterior inferior iliac spine along the arcuate line. The cross section of the proximal part of the second screw inserted into the sacroiliac joint is visible (small arrow). (b) Oblique axial reformatted view of the pelvis CT scan after iliac screw fixation. (c) Oblique axial reformatted view of the pelvis CT scan after sacral screw fixation. This second screw was inserted into the sacroiliac joint to the sacral wing.

The first step of the procedure consisted of percutaneous double osteosynthesis with two perpendicular screws. The optimal position and dimensions of the screws were determined from 3D CT-scan reformations of the pelvis. The percutaneous osteosynthesis procedure was performed under CT guidance with the patient in the prone position under general anesthesia. Skin incisions were made below the posterior superior iliac spine and laterally through the posterior iliac wing toward the sacroiliac joint. Two 11-Gauge trephine needles (TV Laurane Medical, Le Pradet, France) were used to penetrate the bone cortex and define the direction of the screws. The screw path was prepared using two flexible Kirschner wires guided by the pins, and two cannulated screws (DePuy-Synthes Spine system, Raynham, MA, USA) were inserted. The first screw was placed in the iliac bone along the arcuate line and the other from the posterior iliac crest to S1 through the sacroiliac joint (Figure 2). So to insert the distal extremity of the screws into the remaining normal bone, a 7.5-mm-diameter and 135-mm-long iliac screw and a 7.5-mm-diameter and 80-mm-long sacral screw were selected.

**Figure 3:**
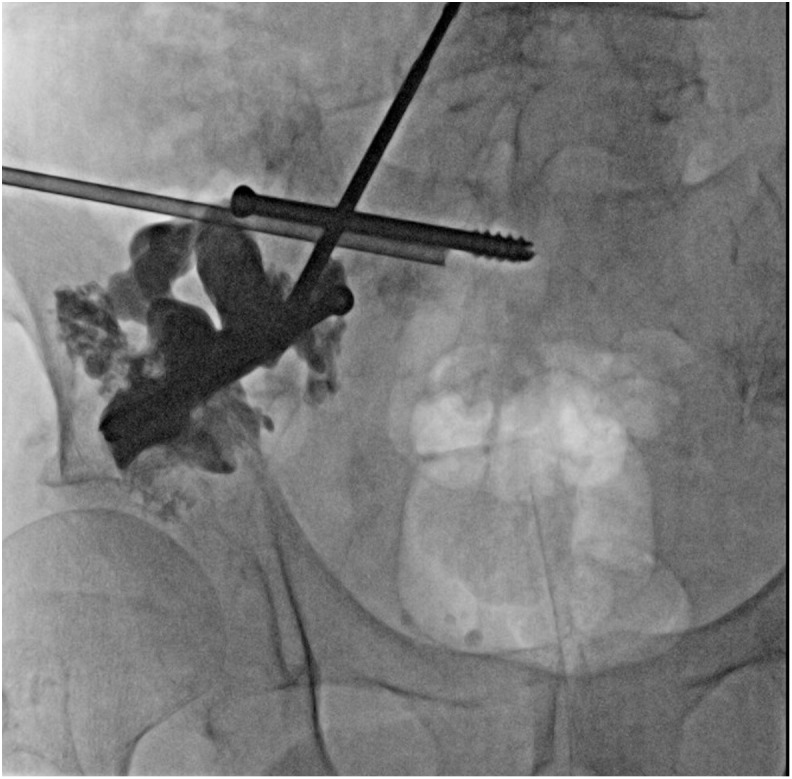
Anteroposterior fluoroscopic view of the right pelvis shows a trephine needle placed parallel to the sacral screw before injection of PMMA cement and after cementoplasty performed along the iliac screw with the same technique (second step of the procedure).

**Figure 4:**
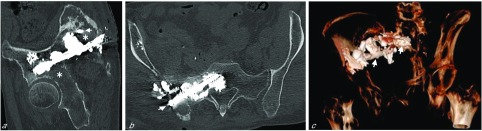
(a) Oblique sagittal reformatted view of post-procedure CT (bone window, slice thickness 1 mm) of the pelvic bones after inserting screws and first step of cementoplasty. The cross section of the proximal part of the sacral screw is visible (small arrow). After this procedure, two large osteolytic areas compromising the whole stability of the osteosynthesis remained, one in the right acetabulum and the other in the right posterior iliac wing with no cement continuity between the two screws at that step (asterisk). (b) Oblique coronal reformatted view of post-procedure CT (bone window, slice thickness 1 mm) of the pelvic bones after inserting screws and first step of cementoplasty shows the sacral screw (small arrow) anchored into the bone with cementoplasty and a slight extravasation of bone cement in soft-tissue. (c) 3D oblique anterior reformatted view of post-procedure CT shows the whole osteosynthesis with the two screws (small arrows) surrounded by cement.

The day after the procedure, with the patient under conscious sedation, the screws were anchored by cementoplasty under fluoroscopic guidance. An 11-Gauge trephine needle was inserted parallel to each of the two screws, and 47 cc of high-viscosity PMMA cement (Vertecem plus, DePuy-Synthes Spine, Raynham, MA, USA) was spread onto the bone alongside the screws (Figure 3). Post-procedure CT showed correct filling of the osteolytic lesions around the screws, with slight cement extravasation but without any consequences for important structures. However, two large osteolytic areas remained, one in the superior acetabulum and one in the right posterior iliac wing, with no cement continuity between the two screws (Figure 4).

**Figure 5:**
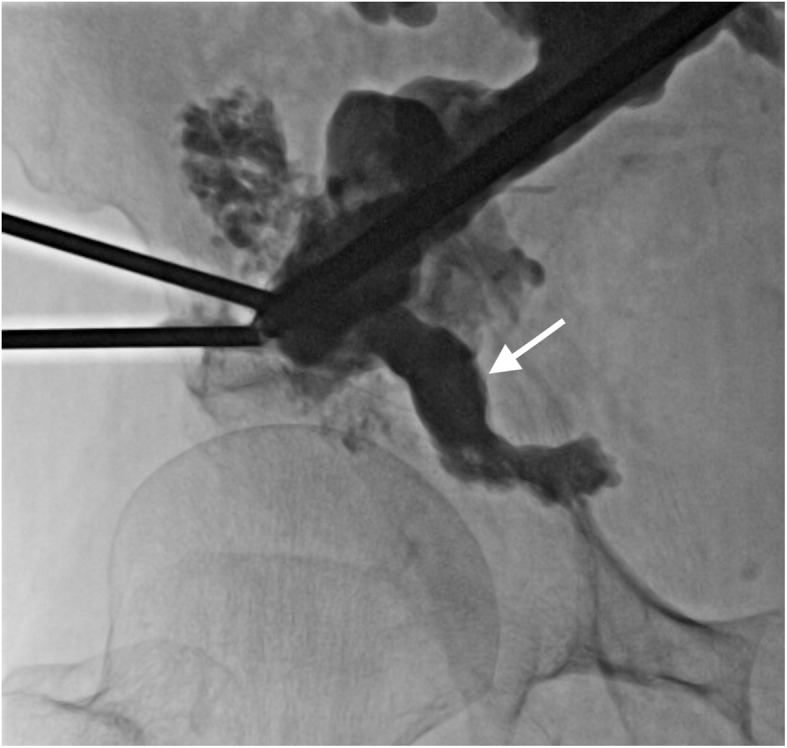
Anteroposterior fluoroscopic view of the right acetabulum filled with PMMA cement (arrow) (third step of the procedure).

**Figure 6:**
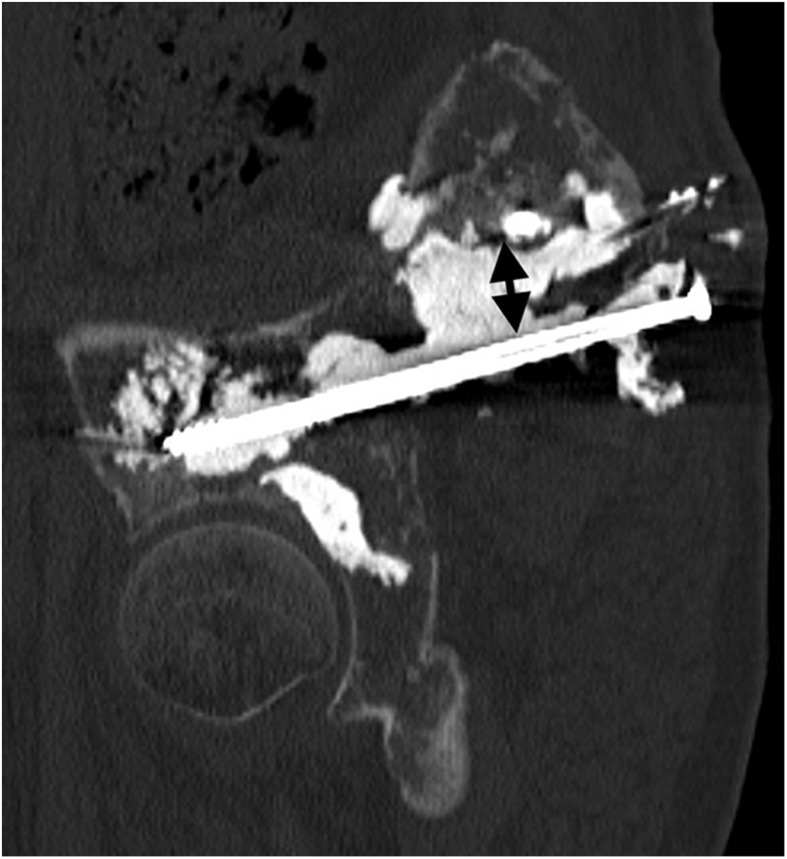
Oblique sagittal reformatted view of the final post-procedure CT (bone window, slice thickness 1 mm) of the pelvic bone shows a continuous cement bridge between the iliac screw and the cross section of the proximal part of the sacral screw (double black arrow).

**Figure 7:**
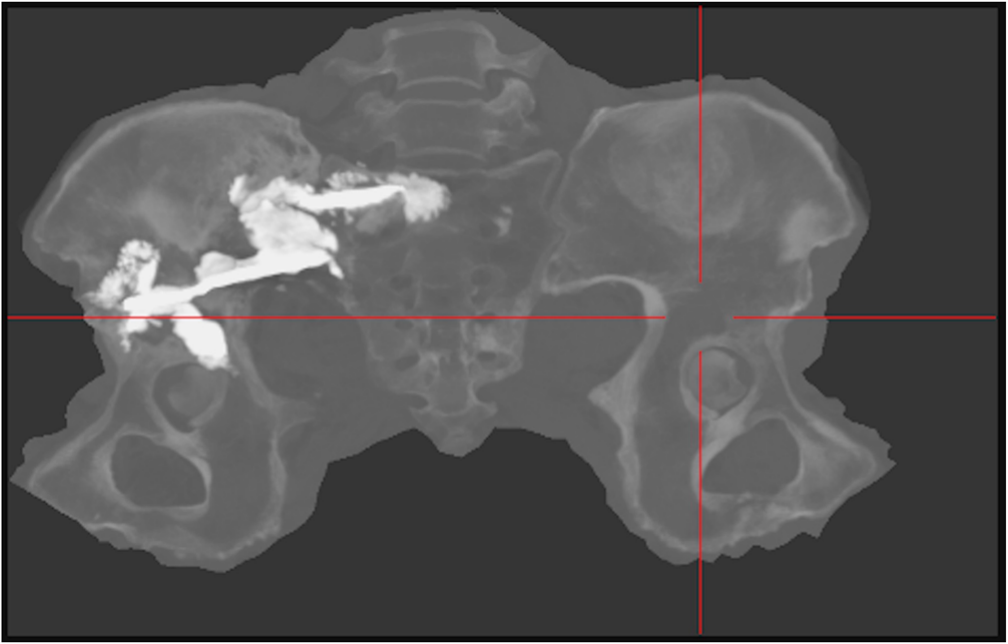
Postoperative CT-scan image with CT Bone Unfolding software representation showing position of the screws into the iliac and sacral bone and their bridging with cement.

Therefore, six days later, with the patient supine and under conscious sedation, 13 cc of the same cement was injected under fluoroscopic control into the acetabulum by an anterolateral approach (Figure 5). One day later, 15 cc of the cement was injected under CT guidance into the posterior iliac wing to create a continuous bridge of cement between the two screws (Figures 6 and 7).

Immediately after the procedure, the patient reported pain relief (VAS 4/10) and was able to stand unaided and walk with one crutch. Analgesic treatment was decreased from 37 μg/72 h to 12 μg/72 h with a fentanyl patch. The follow-up included two courses of systemic chemotherapy.

After two months, the patient presented a superficial abscess at the site of the posterior skin incision around a slight cement extravasation into the subcutaneous tissue. No sign of bone infection was seen on CT scan. The abscess and the extravasated cement were excised. *Escherichia coli* was isolated on bacteriological examination. Cephalosporin antibiotics were administered and continued for 12 weeks. Three months after the procedure, the patient was able to walk without any help. Hip pain was minimal (VAS 2/10) and no pain medication was required. Local infection did not recur. At six months after the procedure, the patient was able to walk unaided almost without pain (VAS 1/10). Unfortunately, ^18^F-FDG PET/CT performed after two courses of chemotherapy revealed bone disease progression. The chemotherapy protocol was changed to a second-line regimen but was not able to control the cancer. The patient finally died from cancer progression eight months after the procedure.

## Discussion

Percutaneous cementoplasty has become the treatment of choice for bone metastases in skeletal areas involving compressive forces such as the vertebral bodies and acetabulum. However, cementoplasty alone is not appropriate to treat bone metastases in areas subjected to shearing forces, such as the metaphysis and diaphysis of long bones and even more so for extensive iliac metastases involving the arcuate line, where high stress is exerted with monopodal standing. Because of the predominance of shearing forces in this skeletal location, the iliac and sacral metastases in our patient could not be treated with cementoplasty alone.

Surgical intramedullary fixation is the reference intervention to restore mechanical stability in osteolytic lesions of long bones. Percutaneous fixation is an alternative technique that may decrease bleeding and operation time and has been used in the sacrum, iliac crest, acetabular roof, pubic ramus, and proximal femur [12,13]. We recently reported a technique of percutaneous nailing with a dedicated locked nail placed along the iliac arcuate line for extensive bone destruction limited to the iliac bone [9]. However, this nail could not be used in the present case because the sacral wing where this nail needed to be locked was completely destroyed as well. Therefore, we needed to adapt the pelvis ring reconstruction technique. In addition to an anteroposterior screw along the iliac arcuate line, a second screw, almost perpendicular, was inserted through the posterior iliac and sacral wing, up to the preserved S1 body, and bone cement was used to create a bridge between the two screws in order to restore the mechanical continuity of the pelvis ring. An advantage of this two-screw technique is that the size and length of each screw can be independently adapted to the anatomy seen on the pre-procedure CT-scan.

Owing to the complete bone osteolysis in our patient, cement augmentation was required to create a mechanical continuity between the two screws and to improve the overall stability of the osteosynthesis. Such extensive lesions cannot be completely filled without hazardous cement extravasation. However, we believe that the cement placement is more important than its amount. To obtain bone competence restoration, the cement must be deposited around the screws in order to anchor the screws into the bone and to prevent their rotation. In addition, in our case, the goal of cementoplasty was also to create a mechanical bridge between the iliac and sacral screws.

At 3-month follow-up, our patient was able to resume walking without any help or need for pain medication. We believe the present case illustrates the concept of using cementoplasty to create a bridge between different screws for increasing the overall strength of the osteosynthesis and to improve pain control [14]. This concept has been illustrated by some reports of treating bone metastases of the femoral neck [5,6] and other long bones [7].

In the present case, four interventional sessions were needed for screw placement and cementoplasty through a double posterior and anterior route. However, with more experience, combined CT and fluoroscopic guidance and adapted devices, the same intervention could be performed in one or two (ifan anterior approach is needed) sessions. We had to insert trephine needles parallel to each of the two screws to improve their bone anchor, but the use of cannulated and perforated screws, which allow for cement injection through the screws, could avoid this step.

CT-scan or fluoroscopic guidance was used for the different steps of the procedure. CT-scan guidance was needed for precise screw placement, whereas fluoroscopic guidance was preferred to control cement extravasation. Therefore, we believe that two of the three cementoplasty sessions could have been avoided using combined fluoroscopic and CT guidance and dedicated cannulated and perforated screws [10].

All interventional sessions were performed percutaneously. Therefore, systemic chemotherapy could be started in the days after the procedure. This is an important advantage of percutaneous techniques over surgery in patients with advanced cancer. External radiation therapy, which is commonly needed to control tumor growth and prevent destabilization, may also be started in the following days.

Percutaneous screwing and cementation were performed before radiation therapy in the present case to prevent fracture and to allow for prompt restoration of the walking ability. The percutaneous procedure, especially the iliac screwing, would not have been feasible in case of pelvic fracture. In addition, repair of osteolytic bone metastases after radiation therapy is not always assured and is often insufficient to restore bone competence depending on the histologic type of cancer. Radiation therapy is feasible after insertion of inert material such as cement or screws, provided dose calculation and delineation are adapted because of tissue density changes [15].

The intervention we report, and especially the injection of cement into the tumor mass without prior tumor ablation, may have induced dissemination of tumor cells into the blood stream. Mohme et al. reported a temporary increase in the number of circulating tumor cells in the peripheral blood 20 min after vertebroplasty or balloon kyphoplasty [16]. However, whether this increase affects the survival of patients with bone metastases is unknown. Also, we do not know whether adding pre-operative radiation or ablation techniques, such as radiofrequency and cryotherapy, could reduce the seeding of viable tumor cells and are valuable treatment options despite their own complications and cost [16].

Although percutaneous, the procedure was complicated by a superficial infection probably related to subcutaneous cement extravasation. The posterior iliac crest is very superficial, with minimal soft tissue thickness, and care should be paid to avoid cement extravasation at that site, as occurred in the present case. Screws inserted at that site should not protrude from the bone and create skin irritation. In addition, our case involved four interventional sessions, and infection may be less likely with fewer interventions.

We present a technique of percutaneous osteosynthesis used with extensive destruction of the pelvis due to bone metastasis. The technique consists of two perpendicular screws anchored into the bone and joined by repeated injections of PMMA cement. The procedure allowed the patient to resume walking unaided and almost without pain.
